# Metabolic profiling of natural and cultured *Cordyceps* by NMR spectroscopy

**DOI:** 10.1038/s41598-019-44154-x

**Published:** 2019-05-22

**Authors:** Yi Lu, Yuee Zhi, Takuya Miyakawa, Masaru Tanokura

**Affiliations:** 1 Department of Applied Biological Chemistry, Graduate School of Agricultural and Life Sciences, The University of Tokyo, 1-1-1 Yayoi, Bunkyo-ku, Tokyo, 113-8657 Japan; 2 Key Laboratory of Urban Agriculture, Ministry of Agriculture, School of Agriculture and Biology, Shanghai Jiao Tong University, Shanghai, 200240 P.R. China

**Keywords:** Metabolomics, Metabolomics

## Abstract

*Cordyceps*, a type of Chinese herbal medicine that exhibits anti-angiogenesis and tumor growth suppression effects, has recently gained increasing popularity. However, high-quality, natural *Cordyceps*, such as *Ophiocordyceps sinensis*, is very rare and difficult to obtain in large amounts. *Cordyceps* is cultured instead of harvested from natural sources, but the quality with respect to the ingredients has not been fully studied. In this study, we performed an NMR metabolic profiling of aqueous extracts of *Cordyceps* without any sample treatment to evaluate the proper species and medium and influence of two different disinfection methods. It was discovered that *Cordyceps militaris* fungus and silkworm chrysalis medium were suitable for cultivation of *Cordyceps*. Furthermore, cordycepin, a *Cordyceps*-specific functional compound, was produced at different growth stages during different cultivation processes, even at the mycelial stage, and was found at three times higher concentrations in cultured *C. militaris* compared to that in naturally occurring *C. militaris*.

## Introduction

*Cordyceps* is one of the most famous plant worms, and it has been used as one of the key materials in various traditional Chinese medicines for almost 2000 years^1^. In recent decades, *Cordyceps* has gained increasing popularity in the world, and several lines of evidence have shown that *Cordyceps* exhibits bioactivities such as anti-angiogenesis effects, tumor growth suppression and the regulation of apoptotic homeostasis^2,3^. Among approximately 400 different species of *Cordyceps*^4^, *Ophiocordyceps sinensis*, *Cordyceps militaris, Cordyceps nutans* and a related species, *Paecilomyces tenuipes*, are widely consumed as health foods and effective traditional medicines in Asia^1,2,5,6^.

*O. sinensis*, one of the most famous and expensive fungal species in the world, thrives in the cold and grassy alpine meadows of Tibet, which is approximately 3000 to 5000 meters high^7^. Therefore, naturally occurring *O. sinensis* is rare and hard to obtain. In recent studies, it has also been shown that *O. sinensis* has anti-oxidant and anti-apoptotic effects, but it is difficult to cultivate^8,9^. *C. militaris*, which is also widely known as a rare caterpillar fungus that is found throughout the northern hemisphere, has similar biological activities to *O. sinensis*, including anti-oxidant and anti-apoptotic effects^1^. Various cultured *C. militaris* are commonly sold as drug materials and health food products in China and South East Asia^10,11^. *C. nutans* is an entomoparasitic ascomycete that parasitizes hemipteran insects, and it has been found in Japan, Taiwan and China^5^. Compared to *O. sinensis* and *C. militaris*, there are only few studies regarding the effects of *C. nutans*. *Paecilomyces* is considered to be an anamorph of *Cordyceps*^2^. *P. tenuipes*, an entomogenous fungi that parasitizes mainly pupae, is a parasitic fungus in the larvae of lepidoptera. Due to its various biological activities, such as the cytotoxic activities of acetoxyscirpenediol and ergosterol peroxide, *P. tenuipes* is highly valuable as a health food and has been used as an effective, traditional, nutritious medicine in China for many years^6,12^.

In association with the rise in popularity, the demands for *Cordyceps* as an herbal supplement and health food have also increased. However, since high-quality, natural *Cordyceps*, such as *O. sinensis*, is very rare and difficult to obtain in large amounts, consumer demand has not been met using natural *Cordyceps*^13^. Although there are some studies on the cultivation process of *Cordyceps*, the actual quality of the cultured *Cordyceps* has not been fully studied in terms of the active compounds^14,15^. This is one of the major barriers to the development of cultivation methods to obtain high-quality cultured *Cordyceps* that can act as a replacement for naturally grown *Cordyceps*. In addition, many components have been found from natural and cultured *Cordyceps* by liquid chromatography (LC), gas chromatography (GC), and capillary electrophoresis (CE) combined with mass spectrometry (MS) and UV-visible spectrophotometry^16–19^, such as cordycepin, adenosine, proteins, amino acids, carbohydrates, carboxylic acids, lipids, glycosides and minerals. However, these conventional methods are all compound-targeted for the specific chemical characteristics of *Cordyceps*^16–19^.

NMR is an analytical technique with a high quantitative ability and reproducibility^20^. NMR spectra are taken in a nondestructive way, and therefore, provides comprehensive information regarding the chemical components directly from complex mixtures in a fast and simple way^21,22^. For the past decade, NMR has been recognized as a powerful technique for discerning the chemical properties of complex mixtures, and it has been widely applied to identify organic compounds in foods, such as milk, yogurt, coffee, mango juice, black garlic^23–27^, and many others. In the present study, solution NMR analysis of water extracts of *Cordyceps* without any separation and purification because consumers usually drink water extracts of *Cordyceps* were performed. Moreover, NMR-based metabolic profiling to investigate and evaluate newly improved cultivation processes of *Cordyceps*, which may bring potential commercial value to the industry of *Cordyceps* cultivation in the future were further performed.

## Results and Discussion

### NMR analysis of natural *Cordyceps* and related species

**Figure 1:**
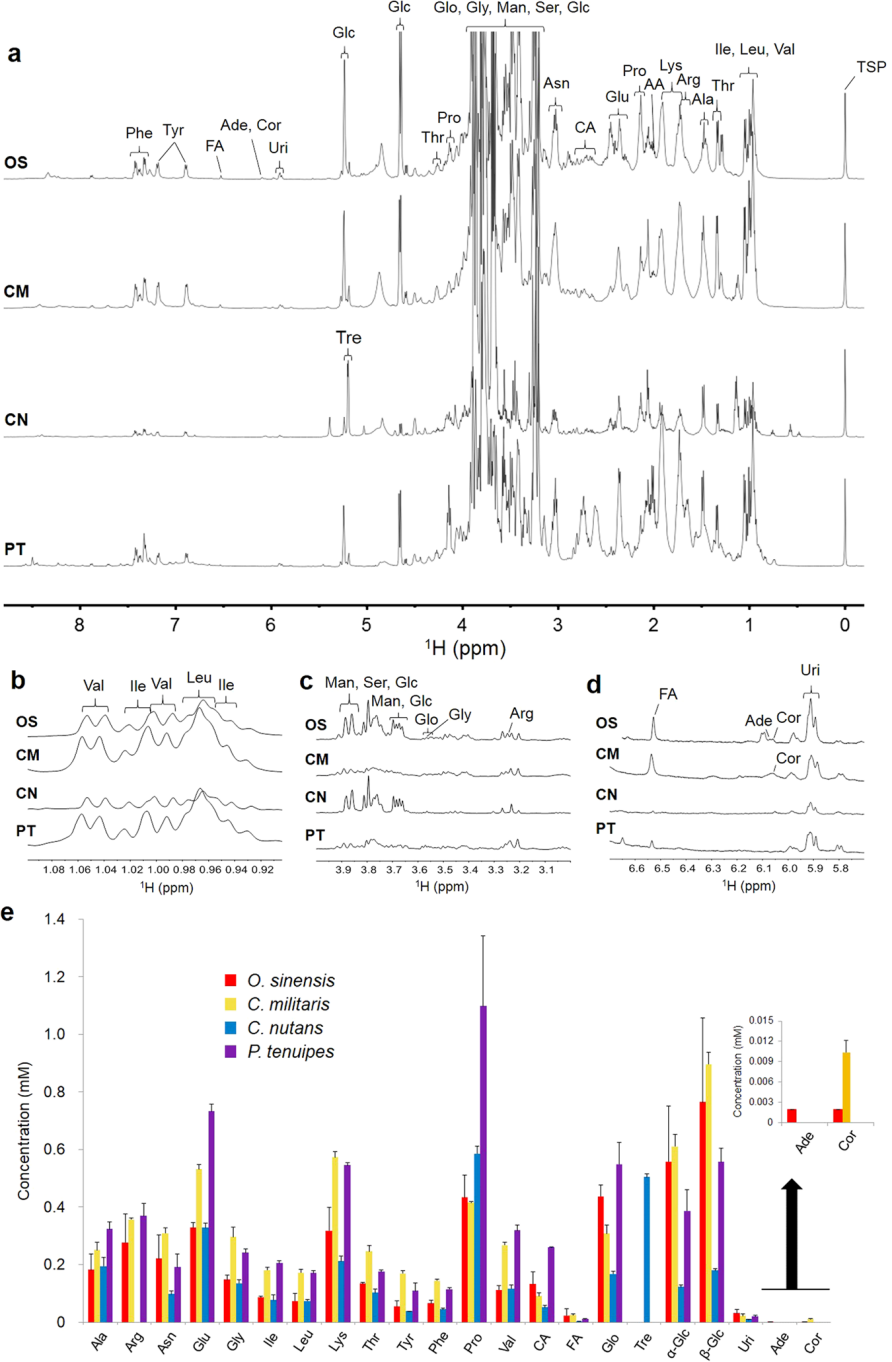
NMR spectral analysis of natural *Cordyceps* and related species. (**a**‒**d**) ^1^H NMR spectra (**a**, −0.2 to 8.8 ppm; **b**, 0.9 to 1.1 ppm; **c**, 3.0 to 4.0 ppm; and **d**, 5.85 to 6.6) of the extracts of *O. sinensis* (OS), *C. militaris* (CM), *C. nutans* (CN), and *P. tenuipes* (PT). (**e**) Concentrations of organic compounds in the extracts of natural *Cordyceps* and *P. tenuipes* determined by NMR spectroscopy. Abbreviations: Ala, alanine; Arg, arginine; Asn, asparagine; Glu, glutamic acid; Gly, glycine; Ile, isoleucine; Leu, leucine; Lys, lysine; Ser, serine; Thr, threonine; Tyr, tyrosine; Phe, phenylalanine; Pro, proline; Val, valine; AA, acetic acid; CA, citric acid; FA, fumaric acid; Glo, glycerol; Man, mannitol; Tre, trehalose; Glc, glucose; Uri, uridine; Ade, adenosine; and Cor, cordycepin. Data are expressed as the means ± standard deviations (SD; *n* = 3).

According to 1D and 2D NMR analysis and spiking experiments, 24 components, including amino acids, organic acids, glycerol and nucleosides, were identified in the extracts from natural *Cordyceps* and *P. tenuipes* (Tables S1–S3). From these, 21 compounds were quantified based on the ^1^H NMR spectra (Fig. 1), while serine, acetic acid and mannitol were not able to be quantified accurately due to heavy signal overlaps. Arginine signals were not detected in extracts of *C. nutans*, whereas trehalose signals were only detected in the extracts of *C. nutans*. Adenosine was only found in the extracts of *O. sinensis*, and cordycepin was not detected in the extracts of *C. nutans* and *P. tenuipes*. This observation suggests that *O. sinensis* and *C. militaris* are the major sources of cordycepin.

Amino acids are regarded as biologically important compounds. For example, muscle protein synthesis is stimulated by amino acid supplementation^28^. 14 types of amino acids were quantified by NMR spectroscopy in the extracts of natural *Cordyceps* and *P. tenuipes*. Citric acid, fumaric acid, α/β-glucose, and glycerol have not been quantified in the extracts of natural *Cordyceps* and *P. tenuipes* in previous studies. Uridine, adenosine and cordycepin were observed by NMR spectroscopy without any separation. Adenosine, which plays a role in sleep control^29^, was specific to the extracts of *O. sinensis*. Cordycepin 3′-deoxyadenosine acts as a nucleoside antagonist, inhibits RNA synthesis^30^, and shows an antitumor effect by inducing apoptosis and cell cycle arrest in human tumor cells^31^. The concentrations of most of the NMR-observed amino acids in *C. militaris* extracts were higher than those in *O. sinensis*. The amounts of glycerol were lower in *C. militaris* than those in *O. sinensis*. Cordycepin, one of the most important biochemicals in *Cordyceps*, showed a 5-fold higher concentration in *C. militaris* than in *O. sinensis*. The concentrations of many of the components in the *C. nutans* extracts were obviously lower than those in *O. sinensis*. Trehalose, which can protect proteins and cellular membranes from inactivation or denaturation caused by a variety of stress conditions in *Cordyceps*^32^, was only found in the extracts of *C. nutans*. Arginine, adenosine and cordycepin were not detected in the extracts of *C. nutans* in the present study. The concentrations of most of the amino acids in the *P. tenuipes* extracts were higher than those in the *O. sinensis* extracts, except for asparagine. Furthermore, the amounts of the four amino acids were also higher in the *P. tenuipes* extracts than the levels found in *C. militaris*, including alanine, glutamic acid, proline, and valine. Generally, natural *O. sinensis* has been proven to be a natural *Cordyceps* with a high quality, but it is hard to obtain in large amounts. We have found that natural *C. militaris* is a good substitute for natural *O. sinensis* in terms of the major ingredients, including cordycepin, since the concentrations of most amino acids were higher than those in *O. sinensis*. Additionally, one of the most important biochemicals in *Cordyceps*, cordycepin, showed a 5-fold higher concentration than that in *O. sinensis*, which was not detected in *C. nutans* and *P. tenuipes* extracts.

### Multivariate statistical analysis of natural *Cordyceps* and *P. tenuipes*

**Figure 2:**
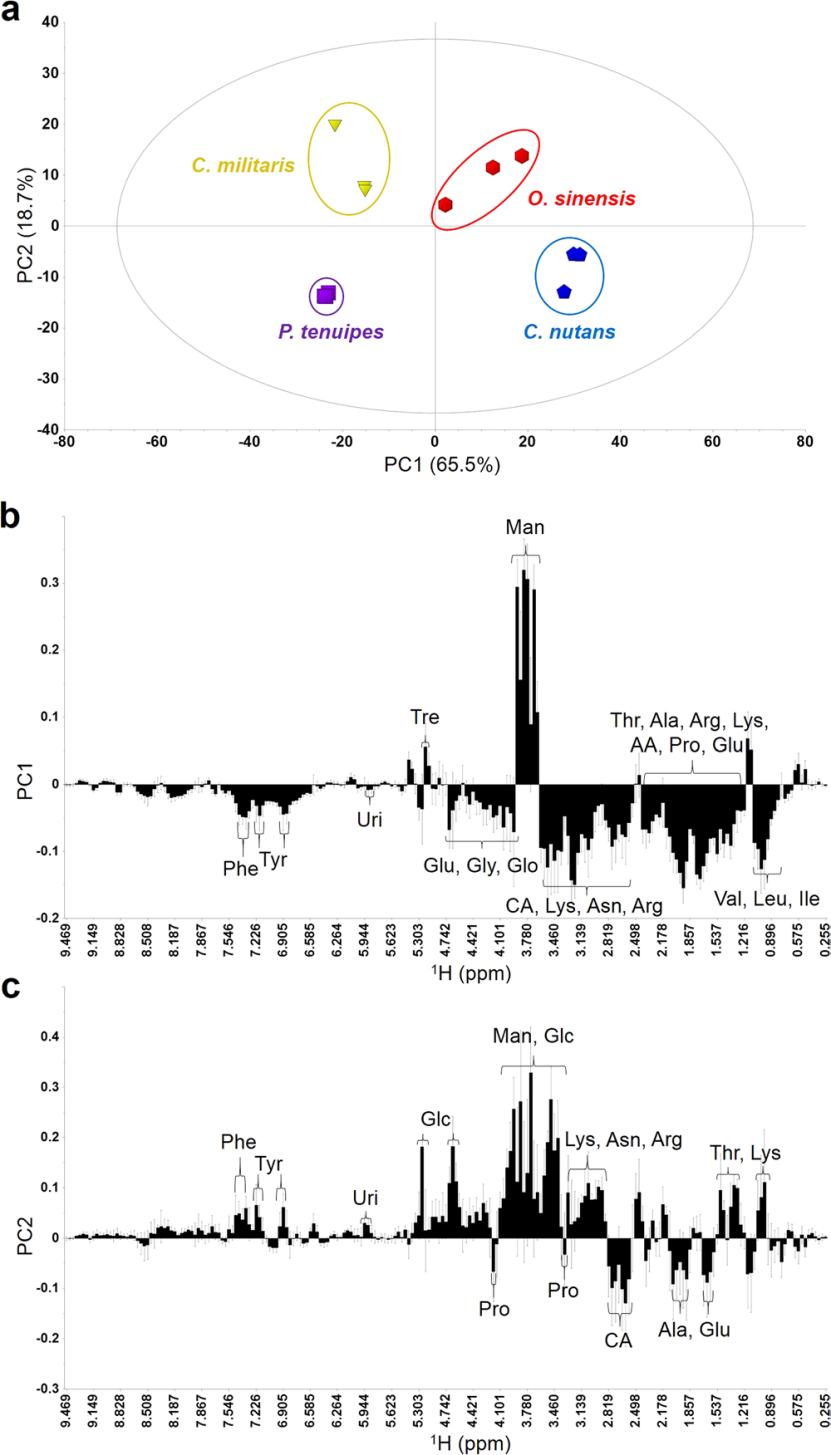
PCA analysis of natural *Cordyceps* and *P. tenuipes*. (**a**) PCA score plot of the extracts of 3 types of natural *Cordyceps* and *P. tenuipes* (*n* = 3). (**b**,**c**) Loading plots of (**b**) PC1 and (**c**) PC2 of natural *Cordyceps* and *P. tenuipes* samples (*n* = 3). Abbreviations: Man, mannitol; and AA, acetic acid. The other abbreviations are the same as those in Fig. 1.

The 3 types of natural *Cordyceps* (*O. sinensis*, *C. militaris*, and *C. nutans*) and *P. tenuipes* were clearly distinguished by the principal component analysis (PCA). The loading plot for PC1 (Fig. 2b) showed that most of the bins had a negative value along the PC1 axis, which means that most of the signals had relatively higher intensities in the extracts from *C. militaris* and *P. tenuipes* than those from *O. sinensis* and *C. nutans*. On the other hand, among the 3 types of natural *Cordyceps* and *P. tenuipes*, trehalose was only detected in *C. nutans* (Fig. 1e) and the signals from mannitol showed high intensities in the extracts from *O. sinensis* and *C. nutans* (Fig. 1c). In Fig. 2c, the results indicate that sugar signals detected in the extracts of *C. militaris* and *O. sinensis* have relatively higher intensities than those in *P. tenuipes* and *C. nutans*, which is consistent with the quantitative data (Fig. 1e). On the other hand, the signals of alanine, glutamic acid, proline and citric acid have relatively higher intensities in *P. tenuipes* and *C. nutans* than in *C. militaris* and *O. sinensis*. Since the signal intensities of *C. militaris* were relatively higher than those of the other natural *Cordyceps* and *P. tenuipes*, along both the PC1 and PC2, *C. militaris* demonstrates great potential as a substitute for natural *O. sinensis*. *C. militaris* was found to contain relatively high concentrations of amino acids, saccharides and nucleosides, including cordycepin, compared to the other types of natural *Cordyceps* and *P. tenuipes* used in the present study.

### NMR spectral analysis of cultured *Cordyceps*

**Figure 3:**
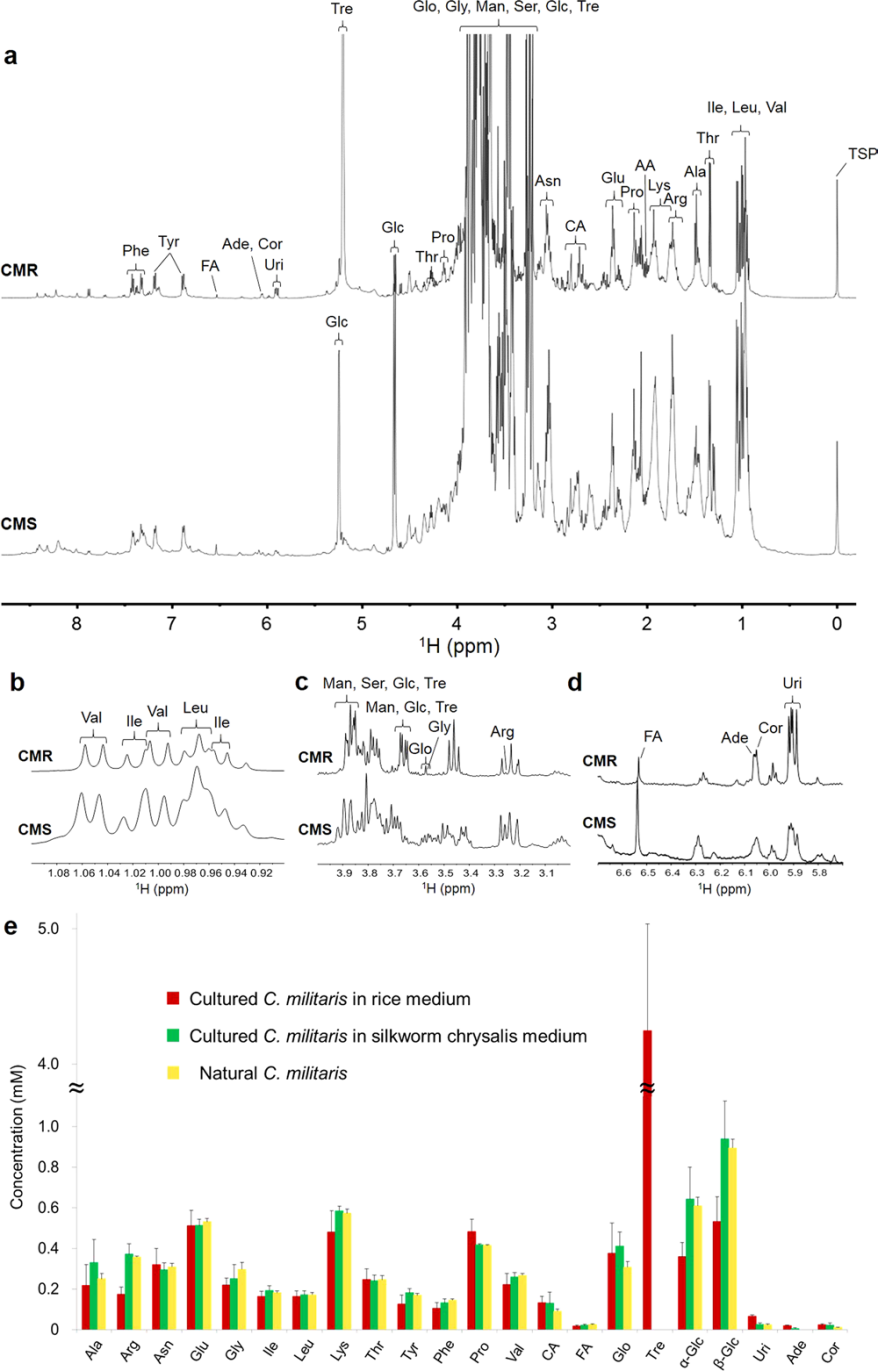
NMR spectral analysis of cultured *Cordyceps*. (**a**‒**d**) ^1^H NMR spectra (**a**, −0.2 to 8.8 ppm; **b**, 0.9 to 1.1 ppm; **c**, 3.0 to 4.0 ppm; and **d**, 5.85 to 6.6) of the extracts of *C. militaris* cultured in a silkworm chrysalis medium (CMS) and in a rice medium (CMR). (**c**) Concentrations of the organic compounds in extracts from *C. militaris* cultured in a silkworm chrysalis medium and a rice medium determined by NMR spectroscopy. The extracts of natural *C. militaris* (*n* = 3) were compared with those of *C. militaris* cultured in a silkworm chrysalis medium and a rice medium (*n* = 5). The other abbreviations are the same as those in Fig. 1. Data are represented as the means ± SD.

Twenty-four components were identified in the extracts of cultured *C. militaris* based on a comparison with the data shown in Tables S1–S3. Then, 21 compounds out of the 24 compounds were also quantified by the ^1^H NMR spectra on the basis of the signal assignments, which are shown in Fig. 3a‒d. The quantitative results for the organic compounds in the cultured *C. militaris* are summarized in Fig. 3e. Trehalose was specific to *C*. *militaris* cultured in a rice medium. The *C*. *militaris* cultured in a silkworm chrysalis medium possessed higher concentrations of α- and β-glucose than that cultured in a rice medium. Trehalose biosynthesis from glucose 6-phosphate is a two-step enzymatic process that involves trehalose-6-phosphate synthase and trehalose-6-phosphate phosphatase, suggesting that the rice medium specifically activates this process^33^. The rice medium is rich in starch, which can be degraded to glucose and induce the synthesis of trehalose by *C. militaris* fungus during the cultivation process.

### Multivariate statistical analysis of natural and cultured *C. militaris*

**Figure 4:**
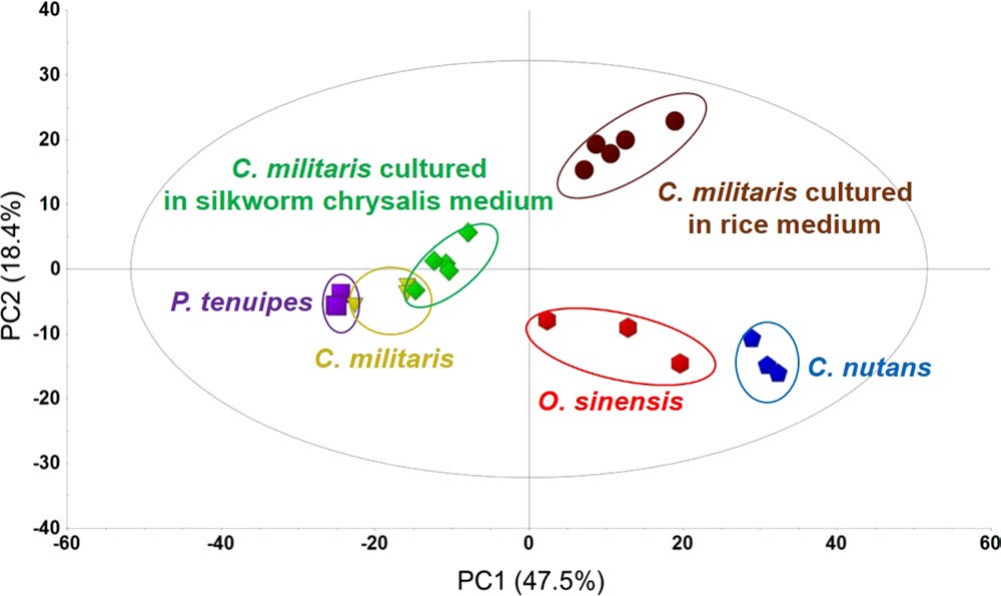
PCA score plot of the extracts of 3 types of natural *Cordyceps, P. tenuipes* (*n* = 3) and 2 types of cultured *C. militaris* (*n* = 5).

A PCA was applied to distinguish the 5 types of natural and cultured *Cordyceps* and *P. tenuipes* and was used to verify whether the two types of cultured *C. militaris* were similar to natural *C. militaris* compared with other related species. As shown in Fig. 4, the samples were clearly distinguished from one another by the PCA score plots. *C. militaris* cultured in a silkworm chrysalis medium showed a distribution more similar to that of natural *C. militaris* on the PCA score plot than *C. militaris* cultured in a rice medium and other natural *Cordyceps*. *P. tenuipes* was also distributed in close proximity to natural *C. militaris*, but the distribution did not overlap with the *C. militaris* cultured in a silkworm chrysalis medium. In general, the silkworm chrysalis medium should be chosen to cultivate *C. militaris*, because the *C. militaris* cultured in a silkworm chrysalis medium possessed almost the same contents as that cultured in a rice medium and it was the most similar to natural *C. militaris*. Thus, the *C. militaris* fungus and silkworm chrysalis medium were suitable for the cultivation of *Cordyceps*.

### Chemical changes in cultured *C. militaris* extracts during cultivation processes

**Figure 5:**
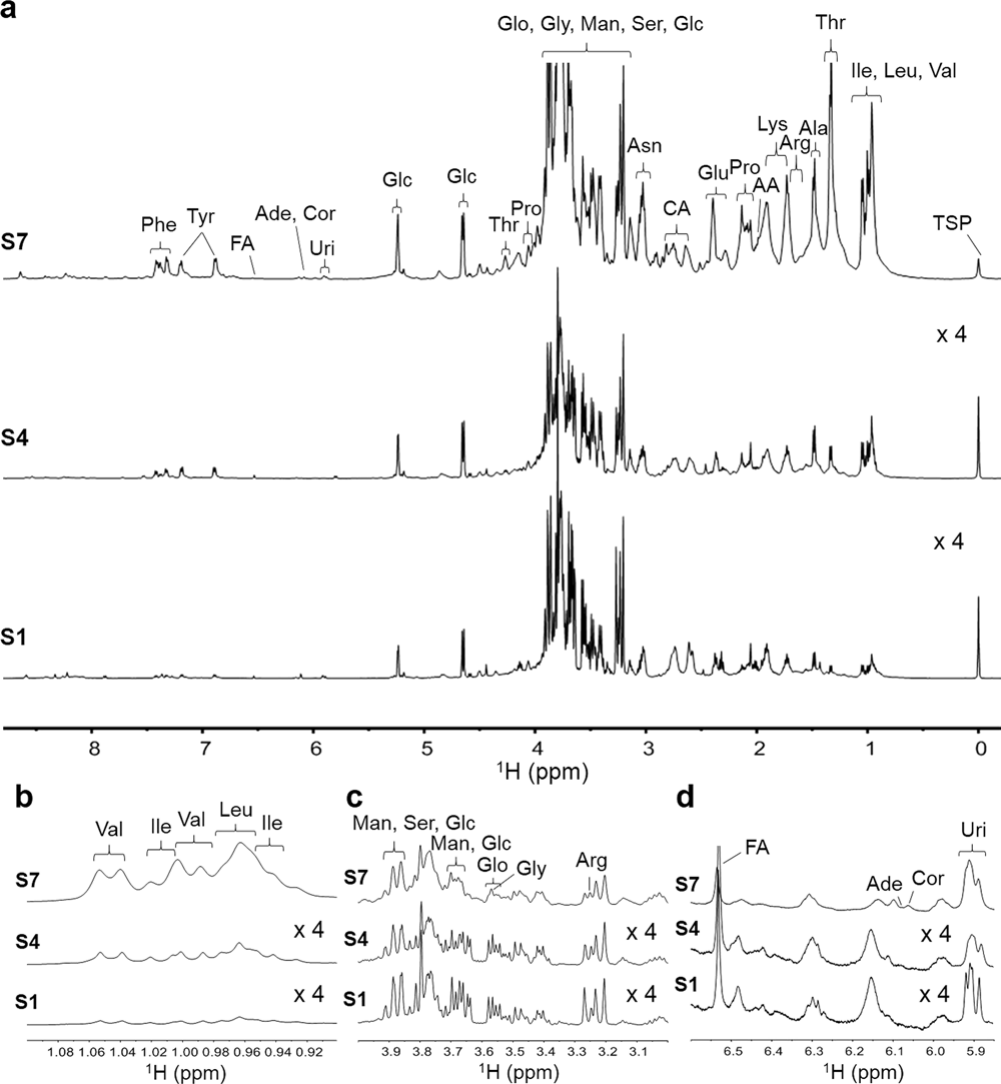
Chemical changes in cultured *C. militaris* extracts during cultivation processes 1. (**a‒d**) ^1^H NMR spectra (**a**, −0.2 to 8.8 ppm; **b**, 0.9 to 1.1 ppm; **c**, 3.0 to 4.0 ppm; and **d**, 5.85 to 6.6) of the extracts of the cultured *C. militaris* samples at stage 1 (S1), stage 4 (S4) and stage 7 (S7) by cultivation process 1. The other abbreviations are the same as those in Fig. 1.

**Figure 6:**
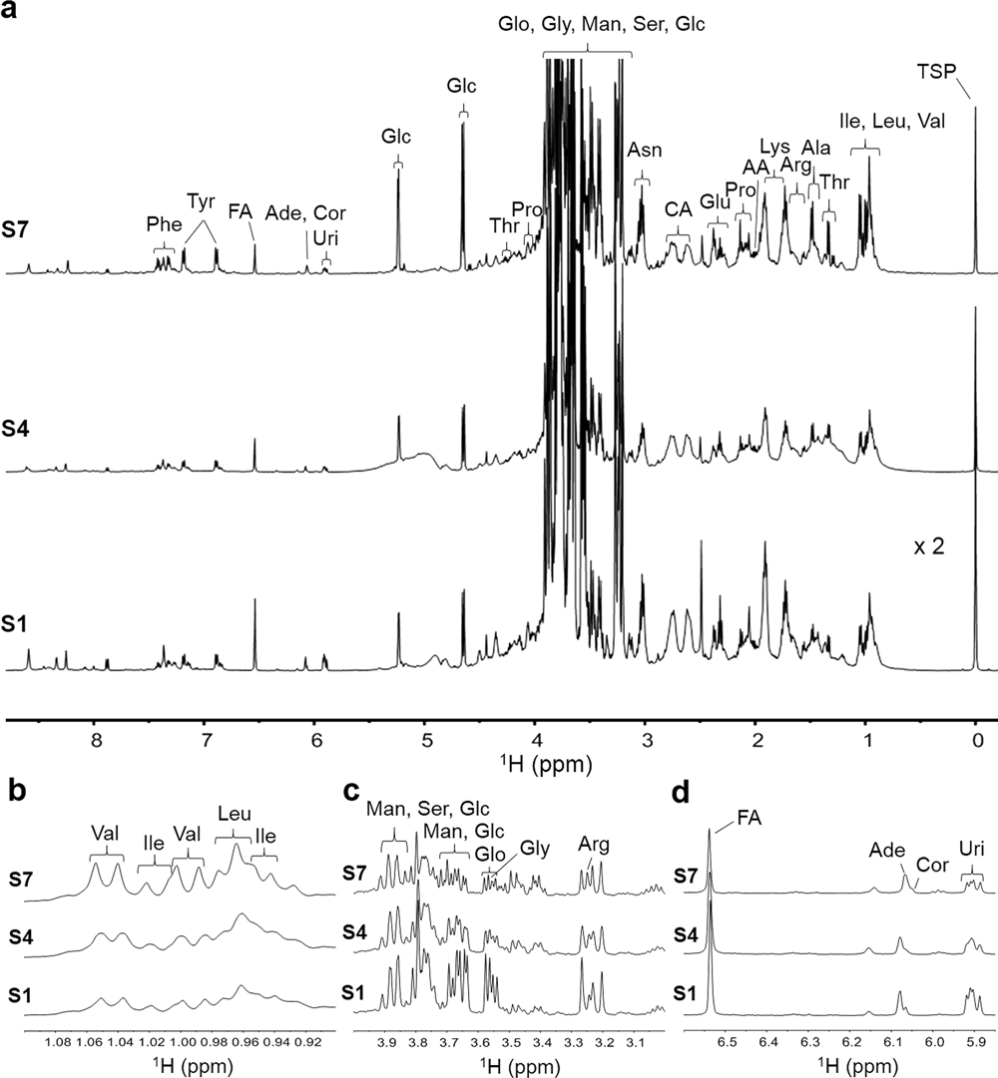
Chemical changes in cultured *C. militaris* extracts during cultivation processes 2. (**a‒d**) ^1^H NMR spectra (**a**, −0.2 to 8.8 ppm; **b**, 0.9 to 1.1 ppm; **c**, 3.0 to 4.0 ppm; and **d**, 5.85 to 6.6) of the extracts of the cultured *C. militaris* samples at stage 1 (S1), stage 4 (S4) and stage 7 (S7) by cultivation process 2. The other abbreviations are the same as those in Fig. 1.

Natural *Cordyceps* may be affected by other contaminated microorganisms, such as fungi and bacteria, in the host worm, which may cause the changes in metabolic processes. However, it remains unclear how the interaction with the contaminated organisms influences the components of the cultured *Cordyceps*. To evaluate it, the chemical changes were monitored during cultivation processes with different disinfection steps. Figures 5 and 6 display the typical ^1^H NMR spectra of the extracts from the *C. militaris* samples culture using two different cultivation processes. The observed compounds include 14 amino acids (alanine, valine, isoleucine, leucine, threonine, arginine, asparagine, lysine, proline, glutamic acid, phenylalanine, tyrosine, glycine, and serine), 3 other organic acids (citric acid, acetic acid, and fumaric acid), 2 saccharides (glucose and mannitol), glycerol and 3 nucleosides (cordycepin, adenosine, and uridine). Among the detectable components, 21 components were quantitatively analyzed because their signals could be separately observed.

**Figure 7:**
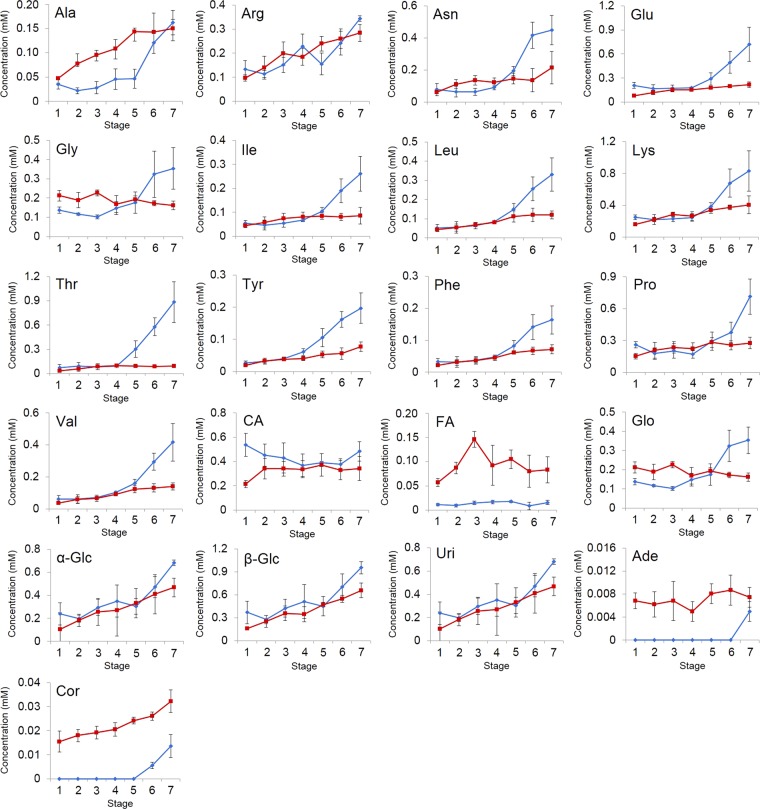
Evolution of the NMR-detected components in the extracts from cultured *C. militaris* samples during two different cultivation processes. Blue lines represent cultivation process 1, and red lines represent cultivation process 2. Abbreviations are the same as those in Fig. 1. Data are represented as the means ± SD (*n* = 5).

**Figure 8:**
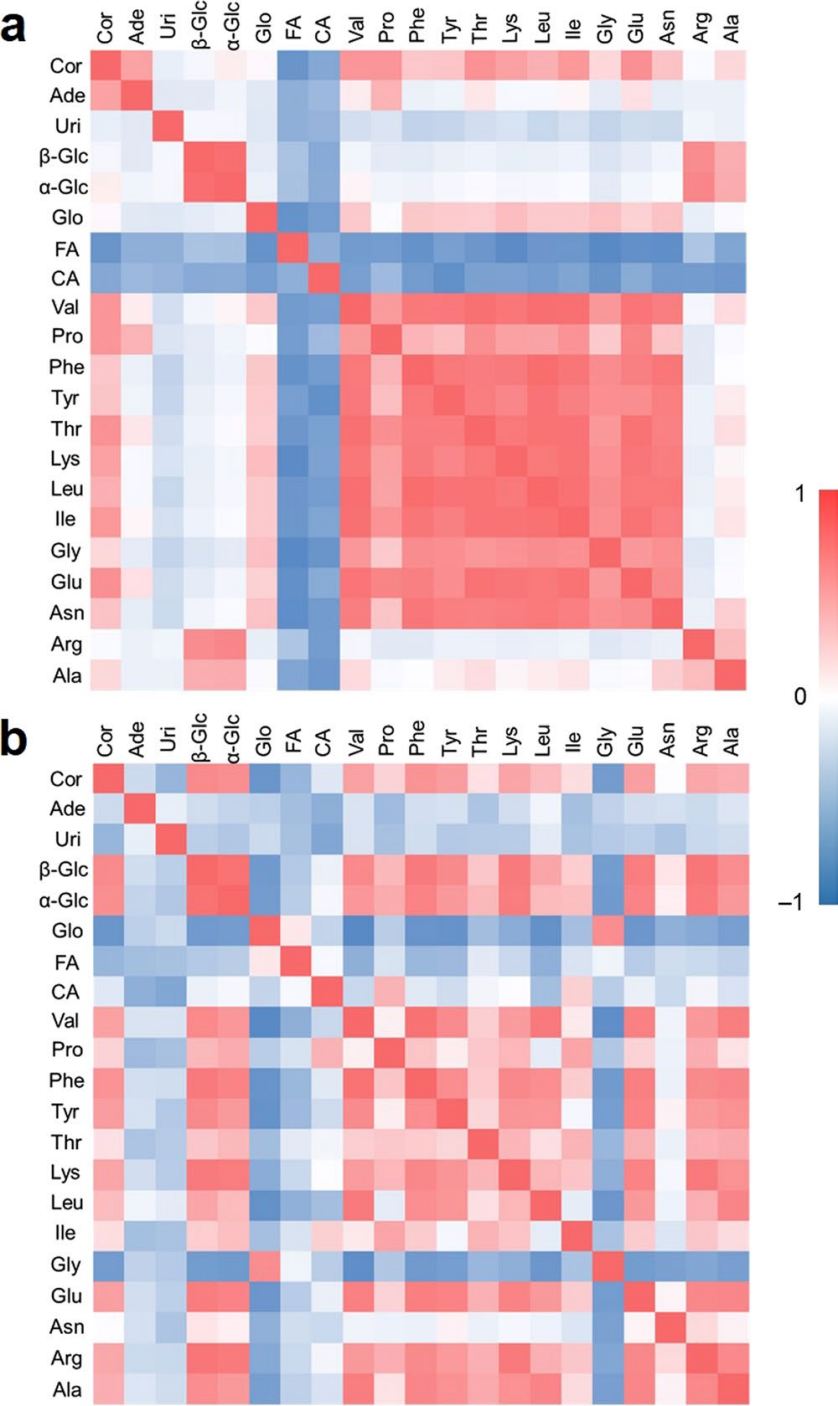
Correlation heatmaps among 21 compounds of the cultured *C. militaris* samples during (**a**) cultivation process 1 and (**b**) cultivation process 2. Abbreviations are the same as those in Fig. 1.

As shown in Fig. 7, the amounts of the 13 types of amino acids observed by NMR, especially from stage 4 to stage 7 during cultivation process 1, were found to be greater. The changes of amino acids were highly correlated with one another (Fig. 8a). Both the concentrations of α- and β-glucose increased during the cultivation processes, maintaining an α/β-anomer ratio of approximately 2:3, showing that α/β-anomer ratio is not affected by the biological processes. Glycerol increased rapidly from stage 5 to stage 7. Uridine tended to increase from stage 5 to stage 7. Adenosine was only detected at stage 7. The signals of cordycepin began to be detected during stage 6, and an increase was observed from stage 6 to stage 7. The pattern of cordycepin was similar to that of adenosine. Compared with the data from cultivation process 1, most of the observed amino acids increased slowly during cultivation process 2. Similar to the chemical changes in cultivation process 1, both the concentrations of α- and β-glucose increased during cultivation process 2, maintaining an α/β-anomer ratio of approximately 2:3. Glycerol decreased slightly during the cultivation process 2. The change of glycerol was negatively correlated with other detected components except for glycine (Fig. 8b). The adenosine signals were detected at stage 1, but the concentration hardly changed over time. The signals of cordycepin were first detected at stage 1, which was a mycelium state, and continued increasing during cultivation process 2 with a 2-fold greater amount compared to that from cultivation process 1. Thus, cordycepin can be synthesized at different growth stages in the cultivation processes with different disinfection steps, even at the stage of mycelia.

### Reasons for the chemical changes during cultivation processes 1 and 2

The mycelium samples were directly disinfected in cultivation process 1, whereas the silkworm chrysalis media were disinfected before seeding of *C. militaris* in cultivation process 2. Cultivation processes 1 and 2 showed different patterns for the production of the amino acids. Based on the results shown in Figs 7 and 8, the amino acids are thought to be obtained via the decomposition of proteins. During cultivation process 1, the relatively higher concentrations of the amino acids were confirmed by comparing the results of cultivation process 2, which indicated that increased consumption of proteins for the production of amino acids occurred in cultivation process 1. Since the silkworm chrysalis medium was not disinfected in cultivation process 1, this observation suggests that the interaction between the *C. militaris* fungus and other microorganisms may cause more consumption of proteins, producing more amino acids during the cultivation process. Glucose was increased during both cultivation processes 1 and 2. Although glucose was thought to be consumed and decomposed into amino acids and organic acids, it can also be produced by polysaccharide degradation, which cannot be observed by NMR spectroscopy. Glycerol is also produced by polysaccharide degradation; however, the concentration of glycerol was not significantly increased in cultivation process 2. Glycerol may be effectively consumed as one of the carbon sources for the growth and/or metabolism of *C. militaris* without the interaction with other microorganisms.

Uridine and adenosine are hypothesized to be produced by phosphoribosyl diphosphate (PRPP), which can be obtained from glucose through the oxidative branch of the pentose phosphate pathway^34^. However, the cordycepin biosynthetic pathway has not been fully revealed. It is thought that cordycepin may be synthesized from adenosine, according to a previous study^35^, and thus the relatively higher amount of cordycepin in cultivation process 2 may be due to the higher amount of adenosine. The relatively lower amount of adenosine in cultivation process 1 may be caused by the influence of other microorganisms, which might consume adenosine as a nutrient source. Since cultivation process 2 included a disinfection method before seeding *C. militaris*, the *C. militaris* fungus could produce cordycepin more efficiently with less interaction with other microorganisms.

### The difference between natural and cultured *C. militaris* products obtained by cultivation processes 1 and 2

**Figure 9:**
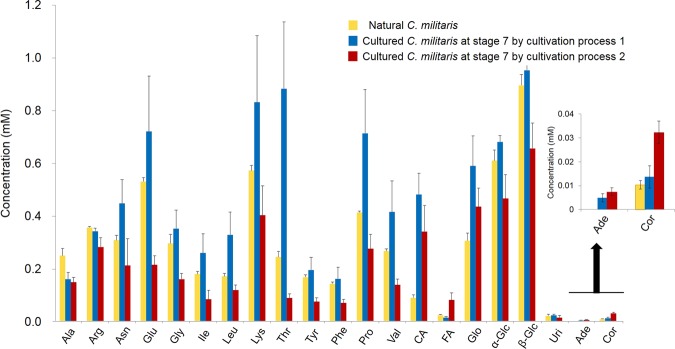
Concentrations of the components in the extracts from natural (*n* = 3) and cultured *C. militaris* samples at stage 7 by cultivation processes 1 and 2 (*n* = 5). Abbreviations are the same as those in Fig. 1. Data are represented as the means ± SD.

Figure 9 shows the concentrations of components in the extracts from the cultured *C. militaris* at stage 7 in cultivation processes 1 and 2 and the natural *C. militaris* samples. In the extracts of *C. militaris* obtained from cultivation process 1, the concentrations of the NMR-detected components, except for alanine, arginine and fumaric acid, were higher than those in natural *C. militari*. In the *C. militaris* extracts produced by cultivation process 2, the concentration of cordycepin was 3-fold higher than that in natural *C. militaris*. These results indicate that the *C. militaris* cultured using cultivation process 1 may be a good substitute for natural *C. militaris* and that cultivation process 2 could be applied to obtain larger amounts of cordycepin, instead of isolation from natural *Cordyceps*.

## Conclusions

The metabolic profiling of natural and cultured *Cordyceps* by NMR spectroscopy to investigate the cultivation processes was performed. It was discovered that the *C. militaris* fungus and silkworm chrysalis medium were suitable for the cultivation of *Cordyceps*. Furthermore, cordycepin can be biosynthesized at different growth stages in different cultivation processes, even at the stage of mycelia, and the *C. militaris* cultured in a silkworm medium that was disinfected before seeding contained three times more cordycepin than natural *C. militaris*. These findings will help further develop cultivation methods for *Cordyceps* for industrial uses and food engineering. The changes in the chemical composition in regarding functional components, such as cordycepin, may also be used to evaluate the influence of *Cordyceps* on human health.

## Materials and Methods

### Samples

Three types of natural *Cordyceps* (*O. sinensis*, *C. militaris* and *C. nutans*) and *P. tenuipes* samples were provided from 3 lots by Cordyce Co. Furthermore, 2 types of cultured *C. militaris* samples, cultured in a rice medium (Shanghai Jiao Tong University) and a silkworm chrysalis medium (Cordyce Co.), were provided from five lots of each type of *Cordyceps* sample from different cultivation processes. The mycelium samples were directly disinfected in cultivation process 1, whereas the silkworm chrysalis media were disinfected before the seeding of *C. militaris* in cultivation process 2. Then, they were transferred into sterilized moisturizing media. The samples of stage 1 were collected soon after disinfection of the mycelium samples, which were referred to as the 0-day state in the present study. The mycelia were incubated at 22 °C, and samples for stages 2 to 7 were collected every 5 days. Samples of the 7 growth stages were freeze-dried before the NMR measurements were taken. The 5 lots were provided for each stage of the *C. militaris* samples.

The *Cordyceps* samples were powdered using a Multi-beads Shocker (Yasui Kikai) with a MSH003(S) holder at 3000 rpm for 10 s. The samples (0.5 g) were soaked in 1.5 mL of deuterium oxide (99.7% Deuterium), and the mixture was shaken for 1 h using a vortex mixer at room temperature. The extracts were centrifuged at 15,000 × g at 20°C for 30 min, and 580 microliters supernatants were transferred to other tubes. Then, 20 μL of 1 mM 3-(trimethylsilyl)propanoic-2,2,3,3-*d*_4_ acid sodium salt (TSP-*d*_4_) in deuterium oxide was added to reach in the final concentration of 33 μM. The pH of the solution was measured with a pH meter. Then the solutions were transferred into 5-mm NMR tubes.

### NMR spectroscopy

NMR measurements were performed at 293 K on an Agilent Unity INOVA-500 NMR spectrometer operating at 11.7 Tesla, observing ^1^H and ^13^C at 499.87 and 125.71 MHz, respectively and was equipped with a 5 mm triple resonance probe. ^1^H and ^13^C NMR chemical shifts are given in ppm related to TSP-*d*_4_ signal at 0.00 ppm as internal reference and coupling constants in Hz.

The quantitative ^1^H NMR spectra were acquired with pre-saturation of HDO signal using a PRESAT pulse sequence as follows: 64K datapoints distributed on a 6,000 Hz spectral width, providing a spectral resolution of 0.183 Hz; acquisition time, 5.461 s; recycle delay, 20 s; and 32 scans. To obtain a proper recycle delay time value, the spin-lattice relaxation time (*T*_1_) was determined using the partial relaxation method^36,37^. The ^13^C{^1^H} NMR spectra were acquired with aid of S2PUL pulse sequence as follows: 64K datapoints distributed on a 32,000 Hz spectral width, providing a spectral resolution of 0.977 Hz; acquisition time, 1.024 s; recycle delay, 2 s; and 100,000 scans. The quantification of the compounds has been performed by directly comparison of the signal area of a no overlapped signal for each compound in relation to the area of TSP-*d*_4_ signal at the concentration of 33 μM. NMR measurements for natural *Cordyceps* and *P. tenuipes* quantitation were performed in triplicate while those for cultured *Cordyceps* in quintuplet. The results in the figures and tables are expressed as the mean ± standard deviation (SD) based on the NMR data. The statistical analysis was performed using SAS University Edition (SAS Institute Inc.). Differences between the means were assessed using analysis of variance (ANOVA) followed by the Ryan-Einot-Gabriel-Welsch (REGWQ) multiple-range test. Differences were considered significant at *p* <0.05.

The ^1^H-^1^H DQF-COSY experiments were obtained by suppressing the HDO signal using the pre-saturation method, and the acquisition parameters were as follows: 32 scans in F2 (2K datapoints) per each 256 increments in F1; spectral widths, 6,000 Hz (F1 and F2); acquisition time, 0.171 s; and recycle delay, 2 s. The ^1^H-^13^C HSQC experiments were generated in the phase-sensitive mode with the following acquisition parameters: 64 scans in F2 (2K datapoints) per each 256 increments in F1; spectral widths, 6,000 Hz for ^1^H and 24,510 Hz for ^13^C; acquisition time, 0.171 s; and recycle delay, 2 s. The ^1^H-^13^C HMBC experiments were measured in the absolute mode with the following parameters: 64 scans in F2 (2K datapoints) per each 256 increment in F1; spectral widths, 6,000 Hz for ^1^H and 32,000 Hz for ^13^C; acquisition time, 0.171 s; and recycle delay, 2 s.

### NMR signal assignments

The preprocessing of the free-induction decays (FIDs) and the subsequent Fourier transformations were performed using the MestReNova software (Mestrelab Research). To assign the signals, some NMR signals were analyzed by comparison with previously published data including NMR assignment data and composition data^38–40^. Although the ^1^H and ^13^C signals of the extracts from *Cordyceps* and *P. tenuipes* were shifted slightly relative to those in the published data due to the use of a different solvent, their correlations were not expected to change in the^1^H-^1^H DQF-COSY, ^1^H-^13^C HSQC and ^1^H-^13^C HMBC correlation maps. The signals were then confirmed and assigned to the candidate compounds on the basis of the ^1^H-^1^H DQF-COSY, ^1^H-^13^C HSQC and ^1^H-^13^C HMBC spectra.

### Multivariate statistical analysis

The ^1^H NMR spectra from 0.20 to 9.40 ppm except HDO signal (4.75‒5.00 ppm) were data reduced by binning process in to equal 0.04 ppm buckets. The buckets area was then calculated and normalized to average sum method. For the multivariate statistical analysis, the resulting datasets were imported into SIMCA-P, version 13 (Umetrics). The quality of the model was described by its R^2^X and Q^2^ values. R^2^X was defined as the proportion of variance explained by the model, indicating the goodness of fit, and Q^2^ represented the predictability of the model^26^.

## Supplementary information

Supporting Information
