# Supplementary material for: Metabolic profiling of natural and cultured Cordyceps by NMR spectroscopy
Source: Sci Rep. 2019 May 22;9:7735. doi: 10.1038/s41598-019-44154-x (PMC6531489; doi:10.1038/s41598-019-44154-x)
Supplement: Supplementary file 1 — Supporting Information [file 41598_2019_44154_MOESM1_ESM.pdf]

Supporting Information

Metabolic profiling of natural and cultured *Cordyceps* by NMR spectroscopy

Yi Lu, Yuee Zhi, Takuya Miyakawa, Masaru Tanokura*

*Corresponding author. E-mail: amtanok@mail.ecc.u-tokyo.ac.jp

Contents

Supplementary Table S1: NMR data (500 MHz, D_2O) for the amino acids in the extracts from *O. sinensis* (pH 6.4).

Supplementary Table S2: NMR data (500 MHz, D_2O) for the organic acids and the nucleosides in the extracts from *O. sinensis* (pH 6.4).

Supplementary Table S3: NMR data (500 MHz, D_2O) for the saccharides in the extracts from *O. sinensis* (pH 6.4).

Supplementary Table S4: Concentrations of the components in the extracts from natural *Cordyceps* and *P. tenuipes* by ^{1}H NMR spectroscopy

Supplementary Table S5: Concentrations of the components in the extracts from cultured *C. militaris* by ^{1}H NMR spectroscopy

Supplementary Table S6: Concentrations of the components in the extracts from *C. militaris* samples cultured using cultivation process 1 at 7 stages of growth

Supplementary Table S7: Concentrations of the components in the extracts from *C.*

militaris samples cultured using cultivation process 2 at 7 stages of growth

Supplementary Figure S1: (**a**) ^{1}H-^{1}H DQF-COSY, (**b**) ^{1}H-^{13}C HSQC, (**c**) ^{1}H-^{13}C HMBC and (**d**) $^{13}C\{^{1}H\}$ NMR spectra of the extract of *O. sinensis*.

Supplementary Table S1. NMR data (500 MHz, D_2O) for the amino acids in the extracts from *O. sinensis* (pH 6.4).

Component	Assignment	^{1}H [ppm] (Multiplicity [Hz])	^{13}C (ppm)
Alanine 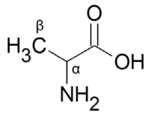	β-CH_3	1.48[a] (*d*: 7.25)	19.08
	α-CH	3.79	53.53
	–COOH		178.69
Arginine 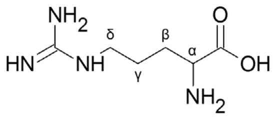	δ-CH_2	3.25[a]	43.30
	γ-CH_2	1.66	26.99
	β-CH_2	1.92	30.64
	$N{=}C(NH_2)_2$		159.72
Asparagine 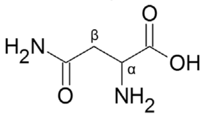	β-CH_2	2.88[a], 2.95[a] (*dd*)	37.41, 54.10
	–$CONH_2$		176.00
	–COOH		177.26
Glutamic acid 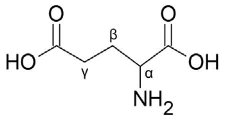	γ-CH_2	2.38[a]	36.00
	β-CH_2	2.11 (*m*)	29.73
	α-CH	3.78	57.43
	α-COOH		184.08
Glycine 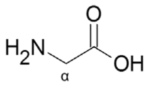	α-CH_2	3.57[a] (*s*)	44.50
	-COOH		175.33
Isoleucine 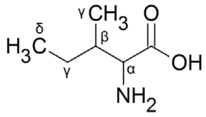	δ-CH_3	0.94[a] (*t*: 7.50)	13.80
	γ-CH_3	1.02 (*d*: 7.30)	17.51
	γ-CH_2	1.26, 1.46	27.48
	β-CH	1.99	39.89
	α-CH	3.63	69.87
Leucine 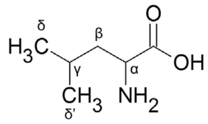	δ-CH_3	0.95[a] (*d*: 5.55)	23.95
	δ'-CH_3	0.96[a] (*d*: 5.30)	25.07
	γ-CH	1.70	26.58
	β-CH_2	1.72	42.61

(To be continued)

Supplementary Table S1. (Continued)

Component	Assignment	[1]H (ppm) (Multiplicity [Hz])	[13]C (ppm)
Lysine	ε-CH_2	3.03[a]	41.95
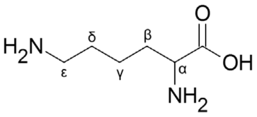	δ-CH_2	1.72 (*m*)	29.27
	γ-CH_2	1.47 (*m*)	24.49
	β-CH_2	1.91	32.90
Phenylalanine	C1 ring		138.04
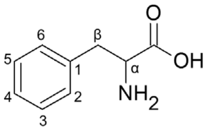	CH-2,6 ring	7.41[a]	132.06
	CH-3,5 ring	7.32[a]	132.28
	CH-4 ring	7.37[a]	130.68
Proline	δ-CH_2	3.34	49.13
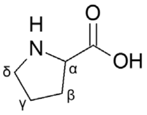	γ-CH_2	2.04 (*m*)	26.63
	β-CH_2	2.05, 2.33	31.86
	α-CH	4.14[a]	64.10
Serine	β-CH_2	3.97	63.21
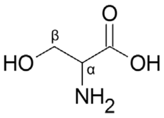	α-CH	3.86	59.42
	–COOH		175.52
Threonine	γ-CH_3	1.33[a] (*d*: 6.50)	22.42
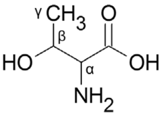	β-CH	4.26	68.87
	α-CH	3.61	63.36
Tyrosine	C1 ring		129.78
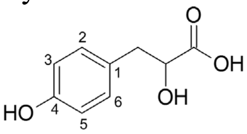	CH-2,6 ring	7.18 (*d*: 7.80)	133.34
	CH-3,5 ring	6.88[a] (*d*: 7.35)	118.72
	C4 ring -OH		157.64
Valine	γ'-CH_3	1.00[a] (*d*: 7.10)	19.77
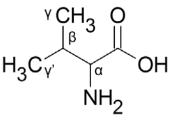	γ-CH_3	1.05 (*d*: 6.95)	20.87
	β-CH	2.28	31.93
	α-CH	3.61	63.27

[a] [1]H signals chosen for the quantitative analysis.

Supplementary Table S2. NMR data (500 MHz, D_2O) for the organic acids and the nucleosides in the extracts from *O. sinensis* (pH 6.4).

Component	Assignment	^{1}H (ppm) (Multiplicity [Hz])	^{13}C (ppm)
Acetic acid	$-CH_3$	1.96 (*s*)	25.75
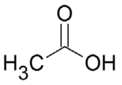	-COOH		183.46
Citric acid	$-CH_2$	2.58, 2.72[a] (*AB*)	47.07
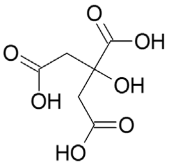	-C		78.56
	CH_2-COOH		181.18
	-COOH		184.04
Fumaric acid	-CH	6.54[a] (*s*)	138.20
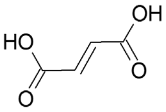	-COOH		177.30
Uridine	CH-1'	5.92[a]	92.18
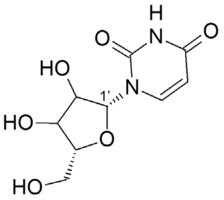	CH=CH	5.89[a], 7.88	105.19, 144.16
Adenosine	CH-2	8.24	155.21
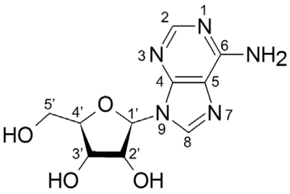	CH-8	8.34	142.48
	CH-1'	6.06[a]	91.22
	CH-2'	4.83	76.34
	CH-3'	4.47	73.31
	CH-4'	4.33	88.53
	CH-5'	3.92	64.19
Cordycepin	CH-8	8.24	155.21
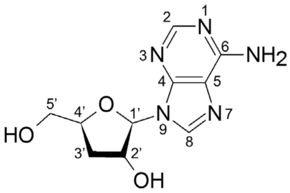	CH-1'	8.32	142.86
	CH-1'	6.07[a]	93.87
	CH-2'	4.85	77.75
	CH-3'	2.27	35.90
	CH-4'	4.63	84.12
	CH-5'	3.83	65.60

[a] ^{1}H signals chosen for the quantitative analysis.

Supplementary Table S3. NMR data (500 MHz, D_2O) for the saccharides in the extracts from *O. sinensis* (pH 6.4).

Component	Assignment	^{1}H (ppm) (Multiplicity [Hz])	^{13}C (ppm)
α-Glucose	CH-1	5.24[a] (*d*: 3.75)	94.96
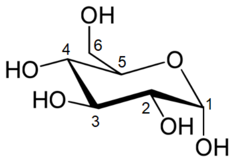	CH-2	3.54	74.48
	CH-3	3.73	75.55
	CH-4	3.42	72.54
	CH-5	3.84	74.29
	CH_2OH-6	3.74, 3.90	63.58
β-Glucose	CH-1	4.65 [a]	98.80
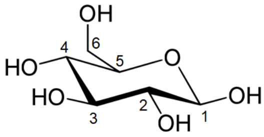	CH-2	3.25	77.04
	CH-3	3.73	75.58
	CH-4	3.42	72.50
	CH-5	3.48	78.53
	CH_2OH-6	3.74, 3.90	63.20
Trehalose	CH-1	5.20[a] (*d*: 3.75)	96.05
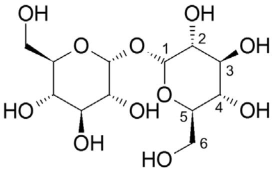	CH-2	3.66	73.88
	CH-3	3.84	75.08
	CH-4	3.46	72.03
	CH-5	3.81	72.10
	CH_2OH-6	3.78, 3.88	63.37
Mannitol	CH_2-1	3.67, 3.87	65.97
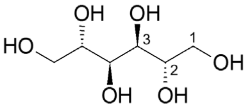	CH-2	3.77	73.65
	CH-3	3.80	72.12
Glycerol	CH-1	3.79	74.79
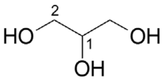	CH_2-2	3.56[a], 3.63	65.40

[a] ^{1}H signals chosen for the quantitative analysis.

Supplementary Table S4. Concentrations of the components in the extracts from natural *Cordyceps* and *P. tenuipes* by ^{1}H NMR spectroscopy.

Component	Concentration (mM)[a,b]			
	Ophiocordyceps sinensis	*Cordyceps militaris*	*Cordyceps nutans*	*Paecilomyces tenuipes*
alanine	0.182 ± 0.054^{A}	0.251 ± 0.027^{B}	0.195 ± 0.030^{A}	0.324 ± 0.024^{C}
arginine	0.277±0.100	0.357±0.004	n.d.	0.370±0.043
asparagine	0.221 ± 0.081^{AB}	0.309 ± 0.018^{A}	0.098 ± 0.012^{B}	0.191 ± 0.044^{B}
glutamic acid	0.328 ± 0.017^{A}	0.531 ± 0.016^{B}	0.328 ± 0.016^{A}	0.733 ± 0.023^{C}
glycine	0.148 ± 0.014^{A}	0.297 ± 0.034^{B}	0.136 ± 0.012^{A}	0.241 ± 0.014^{C}
isoleucine	0.086 ± 0.005^{A}	0.181 ± 0.010^{B}	0.078 ± 0.018^{A}	0.205 ± 0.010^{B}
leucine	0.074 ± 0.026^{A}	0.172 ± 0.011^{B}	0.074 ± 0.005^{A}	0.171 ± 0.008^{B}
lysine	0.319 ± 0.079^{A}	0.573 ± 0.020^{B}	0.212 ± 0.017^{C}	0.546 ± 0.009^{B}
threonine	0.134 ± 0.004^{A}	0.246 ± 0.021^{B}	0.102 ± 0.013^{C}	0.175 ± 0.007^{D}
tyrosine	0.055 ± 0.018^{A}	0.169 ± 0.010^{B}	0.039 ± 0.000^{A}	0.109 ± 0.026^{C}
phenylalanine	0.066 ± 0.010^{A}	0.144 ± 0.006^{B}	0.046 ± 0.004^{C}	0.115 ± 0.005^{D}
proline	0.433 ± 0.076^{A}	0.415 ± 0.005^{A}	0.586 ± 0.026^{A}	1.098 ± 0.244^{B}
valine	0.113 ± 0.014^{A}	0.268 ± 0.009^{B}	0.117 ± 0.012^{A}	0.319 ± 0.018^{C}
citric acid	0.131 ± 0.044^{A}	0.091 ± 0.011^{AB}	0.052 ± 0.006^{B}	0.260 ± 0.002^{C}
fumaric acid	0.024±0.022	0.025±0.003	0.003±0.000	0.011±0.002
glycerol	0.437 ± 0.040^{A}	0.308 ± 0.028^{B}	0.167 ± 0.010^{B}	0.548 ± 0.076^{C}
trehalose	n.d.	n.d.	0.504±0.012	n.d.
α-glucose	0.558 ± 0.193^{A}	0.610 ± 0.041^{A}	0.123 ± 0.006^{B}	0.387 ± 0.073^{A}
β-glucose	0.764 ± 0.291^{A}	0.896 ± 0.042^{A}	0.180 ± 0.006^{B}	0.557 ± 0.048^{A}
uridine	0.032 ± 0.013^{A}	0.023 ± 0.006^{AB}	0.010 ± 0.001^{B}	0.020 ± 0.003^{AB}
adenosine	0.002±0.000	n.d.	n.d.	n.d.
cordycepin	0.002 ± 0.000^{A}	0.010 ± 0.002^{B}	n.d.	n.d.

[a]Concentration was calculated as the mean ± standard deviation (n=3). Different letters in the same row indicate significantly different results (p<0.05).

[b]n.d. represents not detected.

Supplementary Table S5. Concentrations of the components in the extracts from cultured *C. militaris* by ^{1}H NMR spectroscopy.

Component	Concentration (mM) [a,b]		
	Cultured *C. militaris* (in rice medium)	Cultured *C. militaris* (in silkworm chrysalis medium)	Natural *C. militaris*
alanine	0.217±0.103	0.330±0.113	0.251±0.027
arginine	0.173±0.037^{A}	0.372±0.051^{B}	0.357±0.004^{B}
asparagine	0.320±0.081	0.294±0.034	0.309±0.018
glutamic acid	0.511±0.078	0.513±0.031	0.531±0.016
glycine	0.219±0.035	0.251±0.068	0.297±0.034
isoleucine	0.163±0.025	0.193±0.024	0.181±0.010
leucine	0.163±0.029	0.172±0.020	0.172±0.011
lysine	0.481±0.104	0.584±0.024	0.573±0.020
threonine	0.246±0.055	0.240±0.029	0.246±0.021
tyrosine	0.126±0.044	0.181±0.021	0.169±0.010
phenylalanine	0.105±0.027	0.131±0.020	0.144±0.006
proline	0.483±0.061	0.418±0.006	0.415±0.005
valine	0.222±0.055	0.260±0.020	0.268±0.009
citric acid	0.133±0.032	0.129±0.055	0.091±0.011
fumaric acid	0.017±0.006	0.020±0.006	0.025±0.003
glycerol	0.375±0.150	0.412±0.069	0.308±0.028
trehalose	4.246±0.790	n.d.	n.d.
α-glucose	0.358±0.070^{A}	0.643±0.156^{B}	0.610±0.041^{B}
β-glucose	0.533±0.121^{A}	0.938±0.188^{B}	0.896±0.042^{B}
uridine	0.065±0.008^{A}	0.026±0.006^{B}	0.023±0.006^{B}
adenosine	0.020±0.004^{A}	0.006±0.004^{B}	n.d.
cordycepin	0.023±0.006	0.022±0.010	0.010±0.002

[a]Concentration was calculated as the mean ± standard deviation (n=5). Different letters in the same row indicate significantly different results (p<0.05).

[b]n.d. represents not detected.

Supplementary Table S6. Concentrations of the components in the extracts from *C. militaris* samples cultured using cultivation process 1 at 7 stages of growth.

Component	Concentration (mM)[a,b]						
	Stage 1	Stage 2	Stage 3	Stage 4	Stage 5	Stage 6	Stage 7
alanine	0.035± 0.010^{A}	0.022± 0.006^{A}	0.028± 0.013^{A}	0.046± 0.021^{A}	0.047± 0.019^{A}	0.120± 0.022^{B}	0.162± 0.025^{C}
arginine	0.133± 0.036^{A}	0.114± 0.015^{A}	0.151± 0.031^{A}	0.229± 0.050^{B}	0.155± 0.045^{A}	0.242± 0.049^{B}	0.344± 0.011^{C}
asparagine	0.078± 0.038^{A}	0.065± 0.031^{A}	0.065± 0.022^{A}	0.092± 0.016^{A}	0.194± 0.029^{B}	0.417± 0.080^{C}	0.449± 0.091^{C}
glutamic acid	0.209± 0.038^{A}	0.169± 0.047^{A}	0.171± 0.041^{A}	0.178± 0.010^{A}	0.289± 0.077^{A}	0.495± 0.135^{B}	0.721± 0.211^{C}
glycine	0.137± 0.017^{A}	0.117± 0.007^{A}	0.102± 0.011^{A}	0.148± 0.033^{A}	0.176± 0.055^{A}	0.324± 0.082^{B}	0.353± 0.070^{B}
isoleucine	0.054± 0.011^{A}	0.046± 0.021^{A}	0.054± 0.016^{A}	0.068± 0.007^{A}	0.103± 0.017^{A}	0.189± 0.050^{B}	0.261± 0.073^{C}
leucine	0.052± 0.018^{A}	0.055± 0.030^{A}	0.064± 0.016^{A}	0.085± 0.006^{AB}	0.148± 0.030$^{\mathbf{B}}$	0.255± 0.062^{C}	0.330± 0.086^{D}
lysine	0.249± 0.029^{A}	0.220± 0.026^{A}	0.226± 0.031^{A}	0.244± 0.047^{A}	0.383± 0.050^{A}	0.679± 0.177^{B}	0.832± 0.252^{B}
threonine	0.075± 0.039^{A}	0.095± 0.040^{A}	0.085± 0.036^{A}	0.095± 0.011^{A}	0.304± 0.105^{B}	0.579± 0.108^{C}	0.884± 0.252^{D}
tyrosine	0.027± 0.005^{A}	0.032± 0.007^{A}	0.040± 0.006^{A}	0.061± 0.010^{A}	0.106± 0.028^{B}	0.162± 0.024^{C}	0.197± 0.048^{C}
phenylalanine	0.033± 0.010^{A}	0.032± 0.017^{A}	0.037± 0.008^{A}	0.048± 0.003^{AB}	0.083± 0.016^{B}	0.141± 0.039^{C}	0.163± 0.044^{C}
proline	0.259± 0.030^{AB}	0.177± 0.053^{A}	0.202± 0.069^{A}	0.169± 0.036^{A}	0.294± 0.085^{AB}	0.377± 0.096^{B}	0.714± 0.165^{C}
valine	0.060± 0.023^{A}	0.061± 0.027^{A}	0.072± 0.015^{A}	0.102± 0.009^{A}	0.161± 0.025^{A}	0.295± 0.054^{B}	0.416± 0.118^{C}
citric acid	0.536± 0.094	0.452± 0.092	0.430± 0.123	0.368± 0.093	0.391± 0.011	0.378± 0.044	0.483± 0.080
fumaric acid	0.011± 0.003^{AB}	0.009± 0.003^{B}	0.015± 0.004^{AB}	0.017± 0.004^{AB}	0.017± 0.002^{A}	0.009± 0.008^{B}	0.016± 0.004^{AB}
glycerol	0.389± 0.094^{A}	0.297± 0.021^{A}	0.284± 0.050^{A}	0.370± 0.040^{A}	0.396± 0.038^{A}	0.570± 0.042^{B}	0.591± 0.114^{B}
α-glucose	0.237± 0.096^{A}	0.196± 0.031^{A}	0.295± 0.072^{A}	0.349± 0.140^{AB}	0.304± 0.069^{A}	0.471± 0.092^{B}	0.682± 0.024^{C}
β-glucose	0.370± 0.146^{A}	0.277± 0.054^{A}	0.425± 0.117^{A}	0.510± 0.222^{AB}	0.450± 0.126^{AB}	0.701± 0.171^{B}	0.953± 0.080^{C}
uridine	0.012± 0.007^{AB}	0.005± 0.008^{A}	0.009± 0.007^{A}	0.007± 0.008^{A}	0.007± 0.007^{A}	0.014± 0.005^{AB}	0.024± 0.005^{B}
adenosine	n.d.	n.d.	n.d.	n.d.	n.d.	n.d.	0.005± 0.002
cordycepin	n.d.	n.d.	n.d.	n.d.	n.d.	0.006± 0.001^{A}	0.014± 0.005^{B}

[a]Concentration was calculated as the mean ± standard deviation (n=5). Different letters in the same row indicate significantly different results (p<0.05).

[b]n.d. represents not detected.

Supplementary Table S7. Concentrations of the components in the extracts from *C. militaris* samples cultured using cultivation process 2 at 7 stages of growth.

Component	Concentration (mM)[a,b]						
	Stage 1	Stage 2	Stage 3	Stage 4	Stage 5	Stage 6	Stage 7
alanine	0.047±0.003^{A}	0.078±0.020^{AB}	0.096±0.010^{B}	0.109±0.019^{BC}	0.143±0.007^{CD}	0.143±0.039^{CD}	0.150±0.018^{D}
arginine	0.097±0.013^{A}	0.140±0.047^{AB}	0.198±0.048^{BCD}	0.185±0.035^{BC}	0.240±0.030^{CDE}	0.260±0.041^{DE}	0.284±0.036^{E}
asparagine	0.062±0.011^{A}	0.112±0.029^{A}	0.135±0.029^{AB}	0.123±0.027^{AB}	0.146±0.033^{AB}	0.137±0.073^{AB}	0.214±0.101^{B}
glutamic acid	0.081±0.011^{A}	0.121±0.026^{B}	0.155±0.007^{BC}	0.152±0.014^{BC}	0.177±0.017^{CD}	0.196±0.018^{DE}	0.216±0.035^{E}
glycine	0.212±0.028^{AB}	0.189±0.040^{AB}	0.227±0.016^{A}	0.169±0.044^{B}	0.193±0.018^{AB}	0.173±0.014^{AB}	0.162±0.022^{B}
isoleucine	0.044±0.008^{A}	0.058±0.024^{AB}	0.074±0.021^{AB}	0.082±0.018^{AB}	0.085±0.014^{B}	0.081±0.014^{AB}	0.085±0.034^{B}
leucine	0.044±0.010^{A}	0.054±0.018^{A}	0.069±0.010^{A}	0.080±0.008^{AB}	0.111±0.028^{BC}	0.119±0.035^{C}	0.120±0.020^{C}
lysine	0.156±0.012^{A}	0.220±0.061^{AB}	0.281±0.033^{BCD}	0.264±0.055^{BC}	0.339±0.040^{CDE}	0.372±0.030^{DE}	0.404±0.112^{E}
threonine	0.035±0.012^{A}	0.057±0.026^{A}	0.086±0.018^{B}	0.096±0.020^{B}	0.094±0.009^{B}	0.091±0.007^{B}	0.091±0.015^{B}
tyrosine	0.020±0.003	0.032±0.009	0.038±0.004	0.041±0.006	0.053±0.011	0.056±0.018	0.077±0.015
phenylalanine	0.021±0.002^{A}	0.031±0.009^{AB}	0.037±0.012^{AB}	0.045±0.009^{B}	0.062±0.006^{C}	0.067±0.012^{C}	0.072±0.014^{C}
proline	0.151±0.027^{A}	0.208±0.075^{AB}	0.235±0.054^{AB}	0.223±0.054^{AB}	0.281±0.047^{B}	0.255±0.042^{B}	0.277±0.055^{B}
valine	0.036±0.005^{A}	0.058±0.021^{AB}	0.066±0.020^{AB}	0.090±0.008^{B}	0.123±0.022^{C}	0.131±0.026^{C}	0.140±0.022^{C}
citric acid	0.214±0.030	0.342±0.084	0.340±0.059	0.336±0.069	0.372±0.093	0.327±0.078	0.343±0.098
fumaric acid	0.057±0.008^{A}	0.087±0.012^{A}	0.146±0.016^{B}	0.092±0.041^{A}	0.105±0.019^{AB}	0.080±0.033^{A}	0.083±0.027^{A}
glycerol	0.572±0.089^{AB}	0.542±0.125^{AB}	0.650±0.041^{A}	0.495±0.149^{AB}	0.568±0.044^{AB}	0.491±0.037^{AB}	0.436±0.071^{B}
α-glucose	0.104±0.024^{A}	0.182±0.070^{AB}	0.256±0.072^{BC}	0.269±0.069^{BC}	0.331±0.027^{CD}	0.409±0.017^{DE}	0.467±0.090^{E}
β-glucose	0.161±0.009^{A}	0.254±0.076^{AB}	0.358±0.094^{BC}	0.344±0.099^{BC}	0.472±0.028^{CD}	0.547±0.045^{DE}	0.657±0.096^{E}
uridine	0.019±0.004	0.016±0.003	0.020±0.006	0.020±0.005	0.023±0.004	0.024±0.005	0.016±0.008
adenosine	0.007±0.001	0.006±0.002	0.007±0.003	0.005±0.002	0.008±0.002	0.009±0.003	0.007±0.002
cordycepin	0.016±0.004^{A}	0.018±0.003^{A}	0.019±0.003^{AB}	0.020±0.003^{ABC}	0.024±0.001^{CD}	0.026±0.002^{C}	0.032±0.005^{D}

[a]Concentration was calculated as the mean ± standard deviation (n=5). Different letters in the same row indicate significantly different results (p<0.05).

[b]n.d. represents not detected.

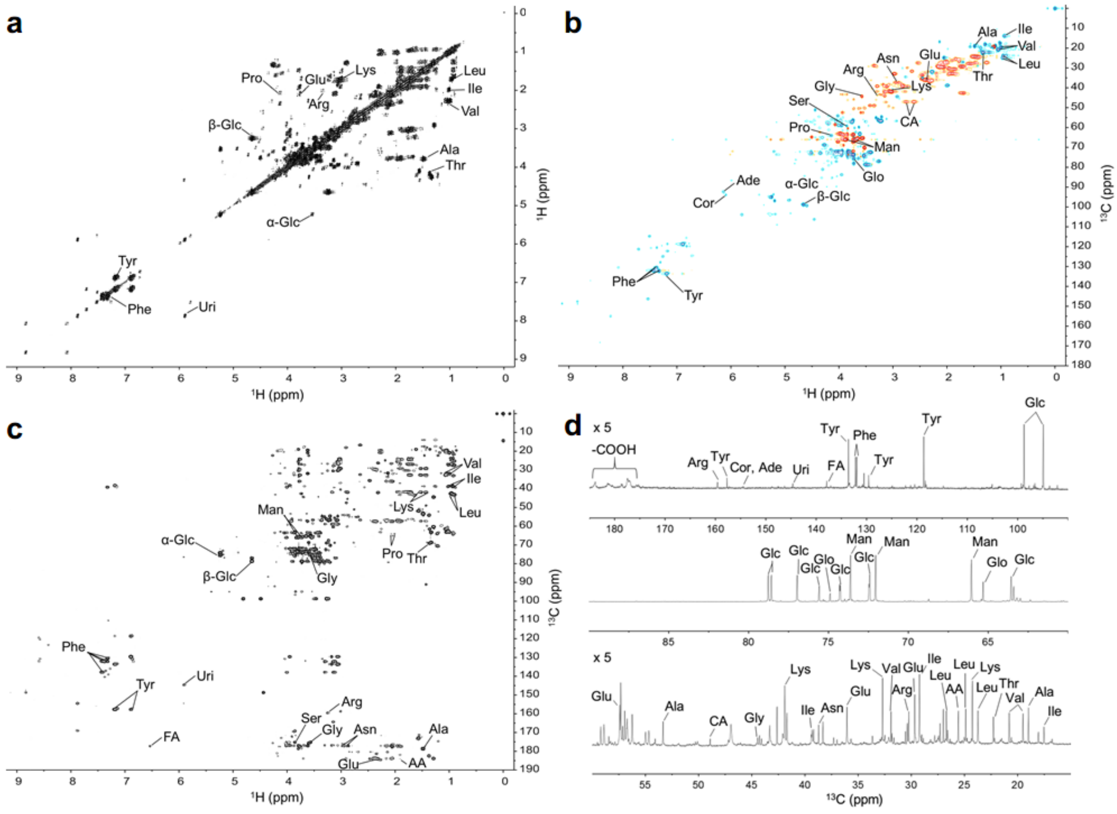

Supplementary Figure S1. (a) ^{1}H-^{1}H DQF-COSY, (b) ^{1}H-^{13}C HSQC, (c) ^{1}H-^{13}C HMBC and (d) ^{13}C{^{1}H} NMR spectra of the extract of *O. sinensis*. Abbreviations: Ala, alanine; Arg, arginine; Asn, asparagine; Glu, glutamic acid; Gly, glycine; Ile, isoleucine; Leu, leucine; Lys, lysine; Ser, serine; Thr, threonine; Tyr, tyrosine; Phe, phenylalanine; Pro, proline; Val, valine; AA, acetic acid; CA, citric acid; FA, fumaric acid; Glo, glycerol; Man, mannitol; Tre, trehalose; Glc, glucose; Uri, uridine; Ade, adenosine; and Cor, cordycepin.
